# Immigration documentation statuses evoke racialized faceism in mental representations

**DOI:** 10.1038/s41598-024-61203-2

**Published:** 2024-05-09

**Authors:** Joel E. Martinez, DongWon Oh, Alexander Todorov

**Affiliations:** 1 Data Science Initiative, Harvard University, Cambridge, MA USA; 2 Department of Psychology, Harvard University, Cambridge, MA USA; 3 Department of Psychology, National University of Singapore, Singapore, Singapore; 4 Booth School of Business, The University of Chicago, Chicago, IL USA

**Keywords:** Immigration, Illegalization, Racialization, Face representation, Reverse correlation, Colorism, Psychology, Human behaviour

## Abstract

U.S. immigration discourse has spurred interest in characterizing who illegalized immigrants are or perceived to be. What are the associated *visual* representations of migrant illegality? Across two studies with undergraduate and online samples (N = 686), we used face-based reverse correlation and similarity sorting to capture and compare mental representations of illegalized immigrants, native-born U.S. citizens, and documented immigrants. Documentation statuses evoked racialized imagery. Immigrant representations were dark-skinned and perceived as non-white, while citizen representations were light-skinned, evaluated positively, and perceived as white. Legality further differentiated immigrant representations: documentation conjured trustworthy representations, illegality conjured threatening representations. Participants spontaneously sorted unlabeled faces by documentation status in a spatial arrangement task. Faces’ spatial similarity correlated with their similarity in pixel luminance and “American” ratings, confirming racialized distinctions. Representations of illegalized immigrants were uniquely racialized as dark-skinned un-American threats, reflecting how U.S. imperialism and colorism set conditions of possibility for existing representations of migrant illegalization.

## Introduction

A principal way the United States has managed immigration is by legislating citizenship and its exclusions. Its imperial relations to other countries created an extractive demand for foreign labor while internal policies restricted access to citizenship and formal inclusion. The combination of nativist and capitalist motives resulted in contradictory immigration policies^1,2^ and in the construction of deportable and illegalized immigrants^3–6^—“illegalized” here reflects how illegality is an *actively imposed* institutional marker of civic exclusion and displacement^7^. Immigrants-as-threats was a common theme within these policies and resulting political discourse^8,9^, especially against illegalized immigrants^10–13^. These threat frames spurred the development of technologies for identifying, regulating, and expelling illegalized immigrants^12,14,15^ and of counter-defenses for and by immigrants^16,17^. This context of threat surveillance and its contestation has likely shaped the visual construction of illegalized immigrants. This project therefore aims to characterize who is mentally visualized when thinking about illegalized immigrants and how illegality is encoded in these representations.

### Mentally constructing illegality

Illegality, like other social categories, emerges from interactions between various actors (e.g., courts, borders, technology, law enforcement, citizens) that make use of contextually-shifting heterogeneous sets of features (e.g., speech, documents, neighborhoods) to classify people and impose consequences onto them^18–22^. These interactions shape mental representations of what illegality is and can be or look like^23^. Mental representations are schemas that facilitate the category construction process as reservoirs of associations that connect people, their features, categories, and consequences together. For instance, support for social policies can depend on beliefs about who the typical beneficiary is^24–26^. Immigration policy support has been linked to the mental organization of “immigrants”, which influences which immigrants become salient^27,28^.

Previous studies examining representations of illegality have focused on *non-visual* feature-category associations^23,29,30^. Nationalities, economic statuses, and occupations are features used to classify others as illegal in the U.S.^23,29–31^ Illegality is suspected when immigrants are presented as immigrating from Syria or various countries in Latin America and Africa, holding low status occupations, or being economically vulnerable^23,29,30^. These racialized feature associations likely reflect the history of illegality becoming an institutionalized status. The Johnson-Reed Immigration Act of 1924 first designated nationality quotas, which differentially restricted movement from countries on the Eastern Hemisphere while solidifying racist nationality hierarchies (in favor of Northwestern European countries)^6^. The Immigration Act of 1965 removed the previous quotas but designated limits on visas from both hemispheres, which restricted movement from countries in the Western Hemisphere for the first time. These numerical restrictions combined with U.S. agricultural labor demands to increase “unauthorized” entries from Mexico^3,12^. The recent rise in illegalized movement from countries in Asia and Central America and decline from Mexico^32,33^ further highlights how the specific features and populations marked by illegality will be dynamic due to domestic and foreign policies and resulting migration and displacement patterns.

### Facing illegality

We investigate the face as another potentially diagnostic yet understudied *visual* feature used in perceptions of illegality. Generally, people make assumptions about social category membership and character from even brief exposure to others’ faces^34,35^, which can have persistent and damaging interpersonal consequences^36–38^. The social importance placed on the face reflects “a certain assemblage of power, a certain politics”^39,40^, therefore the social construction of illegality likely includes processes that impose meanings on others’ faces (i.e., faceism). For instance, mobilizing the face as a canvas for ascribing illegalized status has been codified in immigration enforcement policy and technology. The ruling of *United States v. Brigoni-Ponce* (1975) allowed border agents to use “Mexican appearance” as one (though not only) relevant cue when identifying drivers as illegalized immigrants^20^. Likewise, biometric and identification documents make use of one’s body and face to produce a supposed truth about one’s identity in relation to national security^41^. As a consequence, visibility is a major concern for illegalized immigrants and visual appearances are critical to the operation of surveillance environments in which they manage detectability^20,42^.

Given the historical entanglement of illegality, nationality, and race^43,44^, the theoretical temptation is to predict that the faces perceived as illegal should resemble those of certain categories historically associated with illegality (e.g., as Mexicans, Syrians, Nigerians, latinx people, or Asian people)^6,23,29^. However, categorical associations alone cannot inform when someone might be visually classified as illegal because national and race categories group together a collection of visually heterogeneous people and do not have a factual basis in biology, thus cannot be truly read *from* a face^45,46^. We must instead characterize how these visual inferences are read *into* the face through cues like the restricted range of skin pigmentation and facial features *assumed* to represent different categories^47,48^. The more one’s facial appearance matches a category’s presumed prototypical appearance, the more likely they will experience the consequences arising from being identified as that category^49,50^. Rather than a priori assume relevant facial features from category associations, directly measuring them can inform how individuals may be and often are perceived and treated as illegal regardless of their actual citizenship status, nationality, or racialized identity.

Here, we use face-based reverse correlation to measure mental representations of what illegalized immigrants are thought to look like^51,52^. This technique approximates mental representations by repeatedly superimposing random noise over a face image, thus distorting its features, and having people choose which of the noisy images look like a cued target. The resulting images can reveal facial features relevant to the visual construction of illegality.

Illegalized immigrants are often painted by conflicting portrayals in public discourses, therefore relevant features may include cues of how liked they are as a collective, usually encoded in face representations as affective facial expressions perceived as trustworthy or threatening^53^. The ongoing political framing of illegalized immigrants as a criminal threat^54^ has been met with forms of resistance like speaking back against their (mis)representation^16,55^, highlighting immigrant achievement^17^, adopting humanitarian frames (e.g., “no human being is illegal”), or challenging the presumed validity of citizenship and immigration enforcement regimes^56–59^. The common thread across strategies is a push for humanization. These interventions may have blunted public understandings of illegalized immigrants as a threat, a shift which may be reflected in mental representations^28^.

### Current project

The current project investigates how illegalized immigrants are mentally represented. By using face-based reverse correlation, we assume that representations of migrant illegality can be meaningfully captured from face representations. Two studies tested this assumption while providing insights into representational content. Study 1 visualized representations of illegalized immigrants, compared them to representations of other documentation statuses (i.e., documented immigrants, native-born U.S. citizens), and measured the ethnoracial and evaluative cues perceived in the face representations. Following standard procedure, average classification images of each documentation status were rated. However, considering methodological concerns that ratings of the average classification image can produce false positives^60^, individual classification images were also rated. The faces were rated on four traits previously shown to be important to social cognition and perceptions of immigrants and citizens: dangerous^8^, trustworthy^53^, competent^29^, and American^61^. No other trait ratings were collected.

As an additional test that illegality evokes distinct mental representations, Study 2 investigated whether participants would spontaneously sort the unlabeled faces from Study 1 by documentation status if asked to judge visual similarity. The resulting sorting patterns were then used to identify relative contributions of various facial cues to sorting performance. To the extent that face-based reverse correlation can capture meaningful representations of documentation statuses, we expect both studies to show that trait ratings and similarity sorting by naïve samples differentiate face representations by documentation status.

## Study 1: Mental representations of documentation statuses

Since migrant illegality is a mechanism of exclusion from citizenship, its representation is best contextualized through relative comparisons to representations of native-born citizens and to documented (i.e., legal) immigrants. The latter juxtaposition is frequently made by policies demarcating lawful beneficiaries of public goods and by immigrant- or ethnic-based activism hoping to better their positions by distancing from illegality^11^. The comparison between citizen and immigrant representations identifies features specific to representing immigrants, the comparison between documented and illegalized immigrant identifies features specific to representing illegality.

## Methods

Following the typical two-stage reverse correlation task design, an image generating task was followed by image rating tasks with naïve samples. All experimental protocols in Study 1 and Study 2 were approved by Princeton University IRB #7301. All methods were carried out in accordance with relevant guidelines and regulations. Informed consent was obtained from all subjects for all data collection in Study 1 and Study 2.

### Image generating task

#### Participants

Princeton University students were recruited for course credit (N = 181). Data were collected between November 2017 and May 2018. For details of our exclusion criteria please see Appendix A. The final sample included 150 students, 50 in each condition (M_age_ = 19.7, SD_age_ = 1.81). The gender distribution was 57 men and 93 women. The racialized identity distribution was 79 (52.7%) white participants, 48 (32%) asian participants, 5 (3.3%) black participants, 7 (4.7%) latinx/a/o participants, 8 (5.3%) multiracial participants, and 3 (2%) participants whose race was not listed (we make the deliberate choice to not capitalize race labels as a small linguistic intervention against the reification of race in psychological science^46^). Compared to the 2022 U.S. Census^62^, our sample underrepresented the white (75.5%), black (13.6%), and latinx (19.1%) categories, and overrepresented the asian (6.3%) category. Lastly, on a scale of never (1) to all the time (7), participants reported on average some level of contact with immigrants in their daily life (M = 4.57, SD = 1.93). Less than 5 students reported never having contact. There were no significant sample distribution differences between conditions on gender, self-reported immigrant contact, and age (ps > 0.20).

We acknowledge the limitations associated with undergraduate samples, including concerns about the representations we collected generalizing beyond the Princeton sample. We were able to replicate our findings in a separate online sample from California and Texas a year later—a geographically and socio-politically different region from New Jersey. These data were collected for a separate paper that used the same base face^63^, specific details about collection methods and sample description can be found there. We compare the results from both datasets in Supplementary Fig. S1.

#### Participant measures

The race measure asked participants “What is your race/ethnicity:” and had the following response options (we capitalize these response labels as that is how they were presented to participants): “African American/Black”, “White/Caucasian”, “Latinx/o/a or Hispanic”, “Asian/Pacific Islander”, “Native American”, “Multiracial/ethnic”, “Other”. The gender measure asked participants “What is your gender identity:” and had the following response options: “Female”, “Male”, “Nonbinary”. Unfortunately, these response options conflate sex and gender, therefore we pivot and refer to gender terms as originally intended by this measure, “female” responses as woman and “male” responses as man.

#### Procedure

After completing consent forms, participants were led into a room which contained a single computer and chair. The reverse correlation task began with instructions about the content of the task and how to complete it. Participants were asked to visualize one of three different documentation statuses (i.e., undocumented immigrants, documented immigrants, or native-born U.S. citizens). To minimize definitional heterogeneity, we provided definitions for each documentation status: “Undocumented immigrants live in the U.S. without legal immigrant status”, “Documented immigrants live in the U.S. with legal immigrant status”, “Citizens are born in the U.S.”. While citizens can be naturalized immigrants, we wanted participants to visualize what they perceive to be native-born citizens. Participants were instructed that they would be presented with two images and to press the “0” key if the face on the right looked more like the visualized target and the “1” key if the left face looked like the target. Both images were displayed as 9 cm × 9 cm. Each trial displayed the two faces side by side on the center of the screen with the sentence “Which looks more like a [condition target]?” underneath. Participants completed 770 self-paced trials. After each response there was a 1 s fixation cross before the next trial. Since this was an in-lab sample, more trials can improve the quality of the resulting images^64^. Lastly, participants provided demographic information.

#### Stimulus generation

The base face was a morph of all the male faces from the London Face Database^65^, a publicly available face database that states: "all individuals gave signed consent for their images to be ‘used in lab-based and web-based studies in their original or altered forms and to illustrate research (e.g., in scientific journals, news media or presentations)’". We focused on male's faces to reflect the gendered nature of immigration deportations at the time of data collection: about 85% to 90% of deportations have been latino men^66^. A total of 770 pairs of stimuli (total = 1540 faces) were created by repeatedly superimposing random sinusoid noise over the base face using the rcicr package^67^. Each trial displayed a pair of faces with opposing noise patterns.

#### Classification image generation

Individual and average face classification images were computed using the rcicr package. The general analytic procedure includes averaging the noise patterns of the selected images in the reverse correlation task per participant. This average noise pattern is superimposed on the base face to create participant-level faces. The average noise pattern of all selected images across participants is superimposed on the base face to create the sample-average face.

### Image rating task

#### Participants

To gain an understanding of how representations from each condition were perceived, trait inferences about the average and individual classification images were derived from ratings by an independent group of naïve participants. Ratings were collected from various U.S. samples on Amazon Mechanical Turk. Specifically, we asked participants about how American, competent, dangerous, and trustworthy the person in the resulting reverse-correlated images appeared. We conducted a simulated mixed-effect regression power analysis that quantified power for a main effect of documentation status on ratings using the simr package in R^68^. Given a hypothesized effect size of *f* = 0.77 based on previous studies^69^, we tested the impact of increasing the number of participants from 20 to 200 on power. Our simulation estimated that as low as 20 participants would provide 99.90% power. However, we increased the number to 40 per rating sample to account for inaccuracies in simulation assumptions and likely exclusions from unreliable data in online samples (see Appendix A). We consider unreliability as data from participants that exhibited a test–retest reliability of zero or less (see Procedure for details). The following describes the final samples after exclusions. The three average faces generated from each set of participants in each condition (undocumented, documented, native-citizen) were rated in December 2018 on *Americanness* (N = 40, M_age_ = 40.7, SD_age_ = 13.2, 21 men, 19 women), *competence* (N = 40, M_age_ = 36.1, SD_age_ = 10.6, 20 men, 20 women), *dangerousness* (N = 40, M_age_ = 37.4, SD_age_ = 12.1, 23 men, 17 women), *trustworthiness* (N = 40, M_age_ = 39.3, SD_age_ = 11.6, 24 men, 16 women), and *ethnoracial* category (N = 40, M_age_ = 36.2, SD_age_ = 9.62, 26 men, 14 women).

To mitigate potential inflations of Type 1 error by presenting all the visual heterogeneity contained within the full set of individual faces in each condition^60^, the individual faces were rated in November 2018 on *Americanness* (N = 35, M_age_ = 38.3, SD_age_ = 14, 15 men, 20 women), *competence* (N = 30, M_age_ = 38.4, SD_age_ = 11.2, 16 men, 14 women), *dangerousness* (N = 37, M_age_ = 39.1, SD_age_ = 11.4, 19 men, 18 women), and *trustworthiness* (N = 33, M_age_ = 36.7, SD_age_ = 13.1, 22 men, 10 women, 1 non-binary person). We collected many individual difference measures to better characterize the raters; however, they were not analyzed here. See Supplementary Tables S1 and S2 for descriptive statistics of all participant measures.

#### Participant measures

The race measure asked participants “What is your ethnic/racial identification?” and had the following response options: “Latinx/o/a or Hispanic”, “Black or African”, “White or European”, “Asian/Pacific Islander”, “Indigenous”, “Multiracial”, and “Other”. The gender measure asked participants “What is your gender?” and had the following response options: “Female”, “Male”, “Non-binary/Other”. As mentioned previously, we refer to female and male responses as woman and man responses.

#### Procedure

After providing consent, participants were shown a series of faces one by one and evaluated them on a scale of 1 (not at all) to 9 (extremely) on one of four traits: American, dangerous, competent, or trustworthy. Each face image was presented at 300 pixels × 300 pixels. All trials were self-paced and there was a 250 ms delay before the next trial. For the average classification faces, this included the three faces from each condition in randomized order. For the individual classification faces, this included the 150 faces (50 from each condition) in randomized order. We repeated a random subset of 45 faces (15 from each condition) to assess test–retest reliability (see Supplementary Tables S1 and S2). For each participant, test–retest reliability was defined as a correlation between repeated responses to the same stimuli. During each trial, the face was shown in the center of the screen with the following question underneath “How [trait] is this person?” followed by the scale. To help standardize how “American” judgments were made across raters, we mentioned that the ratings should be based on “how similar to the typical American face” each face is. Therefore, when we refer to “American” judgments or perceived “Americaness”, we refer to perceptions of typicality relative to a presumed American facial prototype. For the race classification task, only the three average faces were shown as it would be a very lengthy task with the full set of individual faces. Eight ethnoracial category sliders were shown below each face, ranging from 0 to 100. Participants rated how likely each face was a member of each ethnoracial category. Target categories were presented as: “Black”, “White”, “American Indian or Native American”, “East Asian”, “Hawaiian or Pacific Islander”, “Latinx/a/o or Hispanic”, “South Asian or Indian”, “Middle Eastern”. The slider values were independent of each other (e.g., each slider could be set to 100). All rating tasks ended with demographic and participant measures.

#### Analyses

Trait and race ratings for the average faces (3 faces rated once) were analyzed using mixed models with maximum likelihood estimation in the lme4 package in R^70,71^. To account for rating dependencies, models’ random effects were maximally specified as justified by the study design^72^. Since each documentation status was represented by only one face, documentation status was a fixed effect while random intercepts were allowed to vary by participant given their repeated measures. However, trait ratings of average faces can inflate Type I error^60^. We therefore focus the main results on ratings of the individual faces (the exception being the race ratings which were only collected for the average faces). Interested readers can find trait ratings for the average images in Supplementary Fig. S2. Trait ratings for the individual faces (150 faces, 50 repeated twice) were also analyzed using mixed models where documentation status was a fixed effect. Random intercepts and random slopes for documentation status were allowed to vary by participant (since documentation status was now represented by multiple faces), and random intercepts were allowed to vary by stimuli and by the interaction between participants and stimuli to account for the repeated measures. Following guidelines for aiding model convergence^70^, we used an iterative optimizer algorithm (bound optimization by quadratic approximation; bobyqa) with the maximum number of iterations set to 500,000. Estimated marginal means, effect sizes (*d*), and confidence intervals for both were calculated from the models and comparisons corrected for false discovery rate using the emmeans package^73^. Degrees of freedom were estimated using the Satterthwaite approximation^74^. Effect sizes were calculated from the mixed models to best approximate Cohen’s *d* by using the sum of the all variance components and residual variance as the population SD^75^.

## Results

### Ethnoracial classifications

**Figure 1:**
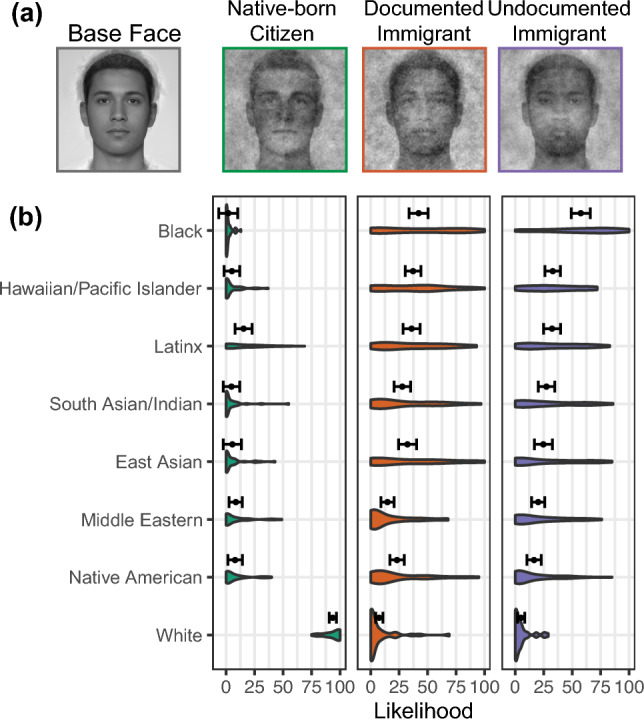
Average classification images and race classifications. (**a**) The base face used in the reverse correlation task, presented alongside with the average classification faces in the native-born citizen (green), documented immigrant (orange), and undocumented immigrant (purple) condition. (**b**) Ethnoracial classifications of the average faces. The x-axis is the perceived likelihood that each face was a member of the ethnoracial categories on the y-axis (sorted by likelihood for the undocumented immigrant face). The violin graphics display the density of the data points. The points and error bars above the violins represent the means and 95% confidence intervals.

The average classification images show highly distinct visual representations for different documentation statuses (Fig. 1a). These visual distinctions occurred along low-level features (e.g., skin color, facial features), which influenced their perceived ethnoracial memberships (Fig. 1b). Every ethnoracial category (N = 8) exhibited a significant main effect of documentation status (range of *Fs*(2,78) = [5.75, 1043.5], range of *ps* = [< 0.0001, 0.005]). The average native-born citizen face was perceived as most likely to be white compared to the average documented immigrant (*b* = 86.1 CI[80.7, 91.5], *d* = 8.43 CI[7.3, 9.6], t(78) = 39.01, p < 0.0001) and undocumented immigrant face (*b* = 88.5 CI[83.1, 93.9], *d* = 8.66 CI[7.5, 9.9], t(78) = 40.1, p < 0.0001). Conversely, both immigrant faces were perceived as more likely to be from all the other ethnoracial categories compared to the native-born citizen face. The largest difference between the undocumented immigrant and the native-born citizen face occurred for the black category (*d* = 2.09 CI[1.60, 2.60]), the smallest was the native American category (*d* = 0.43 CI[0.06, 0.81]). The largest difference between the documented immigrant and native-born citizen face occurred for the black category (*d* = 1.51 CI[1.04, 1.97]), the smallest was the Middle Eastern category (*d* = 0.35 CI[0.03, 0.73]). The only ethnoracial category in which the documented and undocumented immigrant faces were rated significantly differently was the black category. The undocumented immigrant face was perceived as more likely to be black (*b* = − 15.5 CI[− 29.3, − 1.70], *d* = − 0.58 CI[− 1.01, − 0.16], t(78) = − 2.75, p = 0.007). Undocumented-black was also the only rating to exceed 50% likelihood within both the documented and undocumented ratings (M = 57.6 CI[49.2, 65.9]).

To summarize, the native-born citizen face was perceived as mostly white, while the two immigrant faces were perceived as anything-but-white. The undocumented immigrant face was also perceived most likely as black, compared to the other two faces. However, using a more geographically varied face database for the base face could have shifted the pattern of classification differences (see limitations section).

### Trait ratings

#### Individual classification image ratings

**Figure 2:**
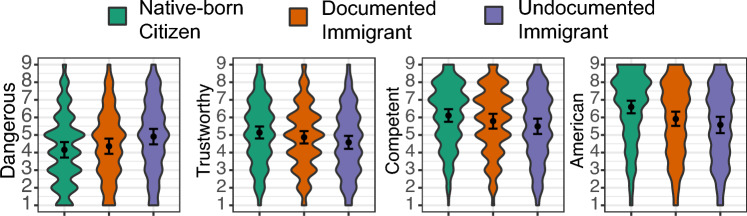
Trait ratings of individual classification images. Ratings of individual classification faces for native-born U.S. citizen (green), documented immigrant (orange), and undocumented immigrant conditions (purple). The violin shapes reflect the density of the rating data much like a sideways density plot. The error bars represent 95% confidence intervals.

Documentation status significantly predicted *American* (*F*(2,101.8) = 14.99, *p* < 0.0001), *competent* (*F*(2,82.3) = 10.56, *p* < 0.0001), *dangerous* (*F*(2,148.8) = 13.46, *p* < 0.0001), and *trustworthy* (*F*(2,107.9) = 5.63, *p* = 0.005) ratings (Fig. 2). The undocumented immigrant faces were on average rated as less *American* (*b* = − 1.02 CI[− 1.49, − 0.55], *d* = − 0.46 CI[− 0.64, − 0.28], *t*(81.3) =  − 5.34, *p* < 0.0001), *competent* (*b* = − 0.62 CI[− 0.96, − 0.28], *d* = − 0.32 CI[− 0.47, − 0.18], *t*(59.5) = − 4.47, *p* = 0.0001), *trustworthy* (*b* = − 0.56 CI[− 0.98, − 0.15], *d* = − 0.28 CI[− 0.45, 0.11], *t*(48.8) = – 3.31, *p* = 0.004), and more *dangerous* than the native-born citizen faces (*b* = 0.75 CI[0.38, 1.12], *d* = 0.37 CI[0.22, 0.51], *t*(148.3) = 4.91, *p* < 0.0001). The documented immigrant faces were only significantly rated as less *American* (*b* = − 0.67 CI[− 1.04, − 0.31], *d* = − 0.30 CI[− 0.44, − 0.17], *t*(127.5) =  − 4.46, *p* < 0.0001) and *competent* than the citizen faces (*b* = − 0.33 CI[− 0.63, − 0.02], *d* = − 0.17 CI[− 0.30, − 0.04], *t*(70.9) =  − 2.62, *p* = 0.011). The undocumented immigrant faces were rated as less *American* (*b* = − 0.35 CI[− 0.69, 0], *d* = − 0.16 CI[− 0.64, − 0.28], *t*(135.9) =  − 2.40, *p* = 0.018), *competent* (*b* = − 0.29 CI[− 0.52, − 0.06], *d* = − 0.15 CI[− 0.25, − 0.05], *t*(142.6) = − 3.09, *p* = 0.004), *trustworthy* (*b* = − 0.29 CI[− 0.59, 0.02], *d* = − 0.14 CI[− 0.27, − 0.02], *t*(148.4) =  − 2.25, *p* = 0.039), and more *dangerous* than the documented immigrant faces (*b* = 0.55 CI[0.22, 0.88], *d* = 0.27 CI[0.13, 0.39], *t*(154.5) = 4.02, *p* = 0.0001).

The patterns of ratings suggest that the undocumented immigrant face was evaluated more negatively on all traits compared to the native-born citizen face. The documented and undocumented faces were rated similarly on evaluations of Americanness and ethnoracial categories, but differently in social evaluations. The documented face was instead rated closer to the native-born citizen face on social evaluations, signifying the legal/illegal distinction occurred in social trait ratings.

## Discussion

The goal of this study was to visually characterize mental representations of illegalized immigrants. We investigated the cues unique to representations of illegality through comparisons with U.S. citizen and immigrant representations. The results show that representations of documentation statuses are differentially racialized. Replicating research suggesting American and white are implicitly associated^61^, we find that the immigrant representations were perceived as likely to belong to every ethnoracial category except white, while the native-born U.S. citizen representation was perceived as likely to only be white. The illegalized immigrant representation was more likely to be perceived as belonging to the black category than to any other category and more than the other documentation statuses. In line with theorizing on the socially reified relationship between darker features and perceived badness or threat^49,76,77^, the illegalized immigrant representations also received more negative evaluations than the native-born citizen and documented representations as less trustworthy and competent and more dangerous. These patterns occurred in the ratings of the *individual* faces suggesting that, despite visual heterogeneity amongst the faces, raters perceived some common facial cues that differentiated representations of documentation status.

Documented immigrants were represented as both distinct from and similar to illegalized immigrants, revealing nuances underlying the legal/illegal distinction. Representations were similar on evaluations of ethnoracial classification or Americanness, but they differed on trait evaluations—documented immigrants’ representations were evaluated as positively as native-born U.S. citizens’ representations. While research suggests that immigrant acculturation physically “whitens” immigrant representations^69^, our results suggest mere legal status does not, but it does produce positive representations.

## Study 2: Sorting unlabeled representations of documentation statuses

To further test that illegality has a unique mental representation, this study sought to understand how distinct the representational boundaries between documentation statuses are. In Study 1, the ratings from participants naïve to the faces’ documentation status conditions suggested that skin color (i.e., pixel luminance) and affective information (e.g., expressions that resemble smiles or frowns that underlie trustworthiness perceptions^53,78^) can operate as cues that distinguish between documentation status representations. However, one could argue that trait rating tasks direct raters’ attention to specific cues related to the documentation status and the trait. For instance, dangerous evaluations could direct raters’ attention to frowns or darker skin. If asked to instead assess overall visual similarity, how well could naïve participants spontaneously sort the faces by documentations status? This would provide converging evidence that there are salient features visualized into representations of different documentation statuses that others spontaneously identify. This study also sought to identify which cues are spontaneously used to sort faces by similarity – those related to certain social evaluations and low-level features related to pixel luminance values (e.g., skin color, high contrast features).

**Figure 3:**
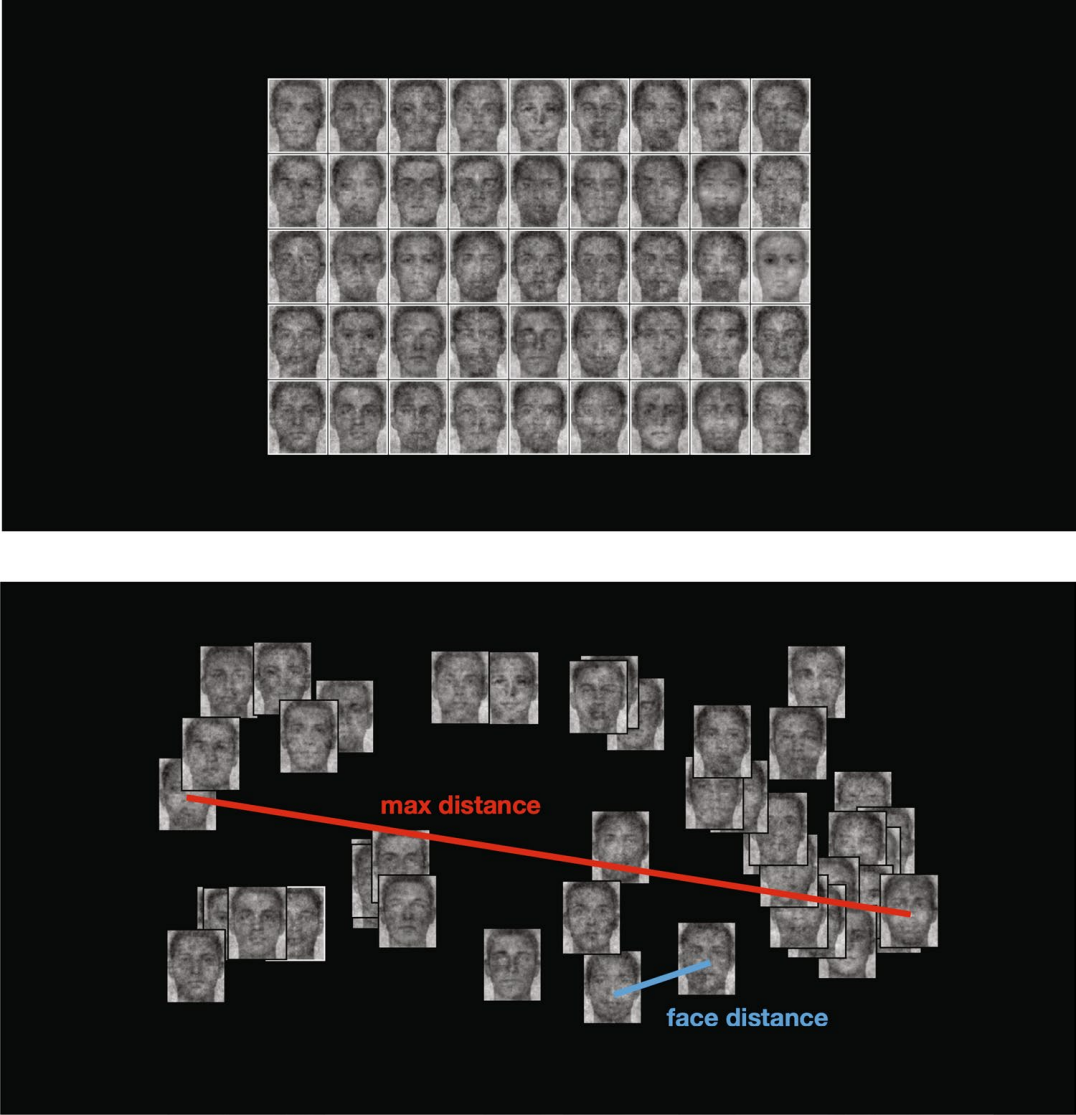
Example of the spatial arrangement task. The top panel is an example screen of the beginning of the task. Faces are arranged in a rectangular format on random locations. The bottom panel reflects an example of what the screen might look like after a participant sorted the faces by similarity. The max distance is the dissimilarity between the two furthest faces on screen (red). The face distance is the dissimilarity between each pair of individual faces (blue).

Participants spatially sorted the unlabeled faces by similarity (i.e., spatial arrangement method^79^). By computing the pairwise distances between the faces in the final sorting pattern (Fig. 3), one can estimate how (dis)similar faces of the same documentation status are perceived to be.

If participants successfully sort the faces, face representations from the same documentation status should be placed near each other and apart from faces from the other documentation statuses. Then, one way to identify the cues that strongly contributed to those decisions is to relate the visual similarity between two faces (i.e., their spatial distance) to the similarity between the same two faces on other measures: trait ratings and pixel luminance values. If a low-level cue, like skin color, facilitates the spontaneous sorting of documentation status, then the pixel similarity between two faces should correlate with their spatial distance. If facilitation instead or also occurs from evaluative inferences made about the faces, then the trait similarity between two faces should correlate with their spatial distance. It is likely that both types of facial cues give rise to face categorization along documentation status, and we can assess their relative contributions.

## Methods

### Participants

The sample consisted of 201 U.S. participants from Amazon Mechanical Turk in November 2018 (M_age_ = 39, SD_age_ = 11.4, 103 Women, 96 Men, 2 whose gender was not listed). See Supplementary Table S3 for the full descriptive demographics. Given the task design, we estimated that 200 participants would provide a large enough sample size to ensure that there would not be any empty face-pair cells (200 participants × 990 unique pairwise combinations from sorting 45 faces = 198,000/11,175 unique pairs from a 150 face similarity matrix = 17.7 participants per cell on average). The resulting average sample size per face pair was 18 (SD = 4).

### Participant measures

The gender measure asked participants “What is your gender?” with the following response options: “Female” (or woman), “Male” (or man), “Prefer not to say”, “Other”. No race or ethnicity measure was collected for this sample.

### Procedure

The spatial arrangement task was implemented using a Qualtrics file provided by the paper introducing the method^79^; the paper provides a step-by-step tutorial, including where the file can be downloaded and considerations for best practices. Participants who accessed the experiment using a screen smaller than 1370 × 768 pixels were directed to a page that informed them they could not participate. After providing consent and reading the task instructions, participants were presented with a black screen that presented 45 faces in the center organized in a 9 row × 5 column grid (see Fig. 3). Participants were instructed that their task was to spatially sort (via drag and drop) the faces by similarity according to the following guidelines: “use the entire screen, place more similar faces closer together, place more dissimilar faces further apart.” Participants could not advance until they moved every face and were given a second chance to change their sorting decisions after clicking “continue” the first time. To make the sorting task tractable, 45 faces were chosen at random from the 150 reverse-correlated faces generated in Study 1, consisting of 15 faces from each condition (native-born citizen, documented immigrant, undocumented immigrant), and randomly placed on the grid. The randomization ensured that different participants sorted some of the same and some different pairs of faces from each condition with the goal that every pair of faces would be seen by a reasonable number of participants. Lastly, participants answered the same demographic and individual difference measures as in Study 1.

### Measures

We constructed six face x face similarity matrices: one for visual sorting similarity, one for pixel similarity, and four for trait similarities (Supplementary Fig. S3).

#### Inter-face visual similarity

The main dependent measure was the spatial distance between each pair of faces. Since the task was presented on a variety of screen sizes (although we ensured that participants could not participate if their screen was smaller than 1370 × 768 pixels), we created a normalized index of interface similarity. The distance between each pair of faces on the screen was considered their dissimilarity. To account for varied screen sizes, for each participant, we divided pair distances by the max distance between any two faces (see Fig. 3). We subtracted these distances from 1 to transform them into similarity scores that ranged from 0 (the max distance any two faces could be) to 1 (completely overlapping on screen). The outcome was a face × face similarity matrix for each participant. Since the instructions did not provide any strategies for how participants should sort (e.g., optimizing cluster boundaries by completely spatially separating face clusters vs. generally placing faces in different areas without attempting to clarify the boundaries), this procedure captures a noisy or conservative measure of category sorting.

#### Inter-face pixel similarity

The pixel similarity matrix was constructed by transforming the pixel luminance values from the noise pattern from each individual-level classification image into a vector, masking out the pixel space external to the contours of the face (which removed the background and kept only the face pixels), and correlating pairs of pixel vectors.

#### Inter-face trait similarity

The trait matrices were constructed by taking the absolute difference of the average rating given to each face in a pair. The rating data from Study 1 (Fig. 2b) were used as the input for these matrices.

### Analyses

#### Similarity by documentation status

If sorting successfully clusters the faces by documentation status, faces *within* a category should be more similar than *across* categories. To test for this pattern, we combined every participant’s similarity matrix while only taking the unique values (i.e., lower triangle). We ran a mixed model where each face pair’s similarity was predicted by their documentation statuses (i.e., a categorical variable indicating the types of the combined pair of documentation statuses of faces, namely, CC = citizen-citizen pairs, DD = documented-documented pairs, UU = undocumented-undocumented pairs, and all cross-category combinations: CU, CD, DU). To account for the repeated pairs at the level of participants and faces, the random effects included random intercepts for participant and for the first face in the pair and separately for the second face in the pair. To aid with convergence, the model was optimized using bobyqa and allowed to iterate up to 500,000 times. Estimated marginal means, confidence intervals, and comparison corrections using false discovery rate from the model were computed using the emmeans package. Degrees of freedom were estimated using the Satterthwaite approximation.

#### Relations between trait similarity, pixel similarity, and sorting similarity

Relations between different similarity matrices were assessed using representational similarity analysis^80^. We computed raw pairwise Spearman correlations between the unique values in each matrix (i.e., lower triangles). However, it is likely that calculating the correlation between any two similarity matrices (e.g., trustworthiness and sorting similarity) will contain information related to the other matrices. To account for this, we also computed partial correlations. By first removing any variance due to the other matrices not currently being correlated and then correlating the residuals, we can get a better measure of the direct relationships between any two matrices while controlling for the rest. Confidence intervals were obtained using the psych package^81^, see Supplementary Table S4.

## Results

### Documentation status sorting similarity

**Figure 4:**
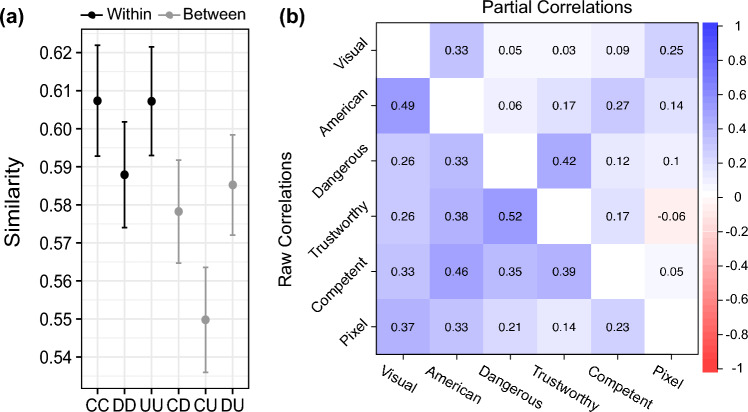
Documentation status visual similarities and relations between trait, sorting, and pixel similarities. (**a**) Average sorting similarity scores between the various condition combinations of native-born citizen (C), documented immigrant (D), and undocumented immigrant (U) faces. In grey are combinations we consider between category (e.g., citizen-undocumented; CU), in black are combinations considered to be within category (e.g., citizen-citizen; CC). Error bars represent 95% confidence intervals. (**b**) Correlation matrix depicting the raw correlations between all the distance matrices in the bottom right triangle, the top right triangle represents the partial correlations. White diagonal are self-correlations.

The face pairs’ combined category label was a significant predictor of similarity scores (*F*(5, 413.1) = 23.37, *p* < 0.0001) (Fig. 4a). Undocumented-undocumented face pairs were more similar than undocumented-citizen (*b* = 0.06 CI[0.03, 0.08], *t*(259) = 7.33, *p* < 0.0001) and undocumented-documented pairs (*b* = 0.02 CI[0.001, 0.04], *t*(189) = 3.15, *p* = 0.004). Likewise, citizen-citizen face pairs were more similar than citizen-undocumented (*b* = 0.06 CI[0.03, 0.08], *t*(279) = 7.34, *p* < 0.0001) and citizen-documented (*b* = 0.03 CI[0.01, 0.05], *t*(230) = 4.04, *p* = 0.0002) pairs. However, documented-documented face pairs were not significantly more similar than documented-citizen (*b* = 0.009 CI[− 0.02, 0.03], *t*(190) = 1.41, *p* = 0.202) or documented-undocumented pairs (*b* = 0.002 CI − 0.02, 0.02], *t*(186) = 0.41, *p* = 0.734). Overall, these patterns suggest that native-born citizen and undocumented face representations contained features that facilitated sorting and that opposed each other (i.e., citizen-undocumented pairings exhibited the lowest average similarity scores), while documented faces were perceived as similar to both native-born citizen and undocumented faces-indicative of a category with visually varied boundaries.

Visual sorting similarity was most related to American similarity (*ρ* = 0.49 CI[0.48, 0.51]), followed by pixel similarity (*ρ* = 0.37 CI[0.36, 0.39]), competence similarity (*ρ* = 0.33 CI[0.31, 0.35]), and the least to dangerous (*ρ* = 0.26 CI[0.25, 0.28]) and trustworthy (*ρ* = 0.26 CI[0.24, 0.27]) similarity (Fig. 4b). The pixel similarity was also most related to American similarity (*ρ* = 0.33 CI[0.31, 0.34]) and least to trustworthy similarity (*ρ* = 0.14 CI[0.12, 0.15]). Strong associations between sorting similarity, American similarity, and pixel similarity suggest that inferences about Americanness elicited by the faces and cues related to pixel luminance (e.g., skin color, highly contrasted facial features) helped participants sort the unlabeled face set by documentation status.

Examining the partial correlations disentangles these relations. The correlation between pixel similarity and American similarity dropped after removing variance due to visual and other trait similarities (*ρ* = 0.14 CI[0.12, 0.16]). The correlation between visual similarity and American similarity also dropped after removing pixel and other trait similarities (*ρ* = 0.33 CI[0.31, 0.34]), yet remained large in magnitude. These findings suggest visual sorting decisions were facilitated by racialized evaluations of Americanness that perceivers imposed on the pixels themselves, rather than by the pixel information alone.

## Discussion

Absent an explicit trait rating task, participants were able to sort unlabeled face representations such that faces who shared a documentation status were more similar than faces across statuses. While we did not directly measure the specific facial features used for sorting, we were able to infer that information using measures from the faces themselves (i.e., pixel luminance) and ratings of the same faces by the raters in Study 1. Supporting our conclusions that mental representations of documentation statuses are racialized, the similarity between faces on their pixel luminance and on their perceived Americanness were highly correlated with their similarity in the sorting task. Such associations highlight the influence of colorism in mental representations of documentation statuses.

## General discussion

We characterized what illegalized immigrants are thought to look like by assessing face representations. With the use of reverse correlation, we found that documentation statuses evoke differently racialized facial imagery. The resulting images are consistent with the racist construction of migrant illegality in the history of U.S. imperialism^6,43,44^. Visualizations of illegalized immigrants were perceived to be non-white (or more so black), less American, trustworthy, competent, and more dangerous than visualizations of documented immigrants and native-born U.S. citizens. We used a spatial sorting task^79^ as a new data-driven way to assess representational boundaries between images from different conditions in reverse correlation studies. Performance on this task showed that the above cues were robustly perceived as participants were able to sort unlabeled face representations by documentation status. The native-born citizen and undocumented immigrant faces were sorted into their own respective categories. This sorting performance was facilitated by the social information (i.e., American-ness) perceived in the visual information held in the faces (i.e., pixel luminance). We supplement the scholarship on the material racialization of illegalized immigrants (e.g., deportation, incarceration) by describing the accompanying racialization that occurs in minds.

### Representing illegality

Illegality is often associated with Mexicans and latinx people more generally^23,29^, while the captured visualization of “undocumented” immigrants was perceived most likely to be black. There are a couple of possibilities for why this pattern emerged. The first assumes that face representations directly reflect nationality or race categories: maybe the association between Mexican/latinx-illegal has been replaced such that the darker-skinned face visualization reflects immigrants from countries perceived as majority black (e.g., Nigeria or Haiti). This possibility relies on bio-essentialist assumptions about what Mexicans look like (i.e., not dark skinned) and the range of nationalities that darker skin could signal^46^. The second possibility instead acknowledges phenotypic variation exists in every nationality and race category^46,50^: if one is thinking of a Mexican when visualizing illegalized immigrants, they are simply not thinking about lighter-skinned Mexicans. A third option is that the representation reflects an abstracted darker-skinned person without a specific national origin (e.g., a Global South prototype). These possibilities all highlight the role of colorism in representations of migrant illegality- visual logics operate in tandem with categorical associations. Future research should clarify the relationship between categorical and visual representations of illegality.

### Where are these representations produced?

Migrant illegality is reified by a complex web of institutional actions and actors (e.g., laws, courts, judges), public and legislative discourses (e.g., threat propaganda, criminalizing policies), objects (e.g., visas, passports), geographies (e.g., borders, segregated neighborhoods), and social practices (e.g., surveillance, deportation). We consider some potential sources that could produce the visual face representations we identified in this study.

Attitudes towards a social category are often theorized through intergroup contact^82^. Illegalized immigrants *are* included in their communities through discretionary and contradictory laws or enforcement practices at national, state, and local levels^2,83^. However, this does not guarantee that contact with illegalized immigrants is extensive or even registered as contact unless they disclosed their status—self-reported measures of contact with illegalized immigrants suggest it is perceived as a rare occurrence (see Supplementary Table S1, S2, and S3). Understandings of illegalized immigrants are therefore less likely to develop from direct social interactions and more from other readily available sources, such as media portrayals.

Our university sample was collected while there was campus activism that reached the highest courts of the U.S. alongside local storytelling from illegalized immigrant students^84^. Positive media portrayals of illegalized immigrants were available on the campus environment. Yet, the sampled students largely visualized illegalized immigrants as relatively dangerous, incompetent, and untrustworthy. Some possibilities for this mismatch are that attention was not paid to these stories, or that local discourse subtyped specific illegalized immigrants as good (i.e., DACA recipients), leaving perceptions of the larger category (i.e., undocumented immigrants) intact.

Another possibility is that criminalizing media at the national level overpowered local discourse, a single criminal portrayal can taint perceptions of an entire group^85^. Despite consistent evidence that immigrants do not increase crime^86^, news coverage of immigrants tends to be overwhelmingly crime-related^8,87^. This media apparatus that produces the “criminal immigrant narrative” also psychologically embeds immigrants into racial hierarchies^28^. In support of this idea, representations collected from an online sample located in border states (i.e., California and Texas) showed a highly similar racialized representation of illegalized immigrants as those from our university sample^63^ (Supplementary Fig. S1).

Official government statistics about criminal behavior and demographic characteristics are yet another publicly available source that can set expectations about illegalized immigrants^12,17,28^. However, the enterprise of statistically characterizing illegalized immigrants is fraught. Initial attempts at enumeration were often mere guesses that contributed to constructing illegalized immigrants as a growing problem^12^. Immigrants’ avoidance of system legibility also disrupts numeric estimates^42^. Criminality statistics have been inflated to an unknown extent by laws that expanded the scope of criminality and deportability to include lesser infractions and retroactive charges^13^. Official statistics may therefore reflect how incarceration, racialization, and illegalization are intertwined in population control projects^88^, rather than characterizing the behavioral reality of a category with continuously shifting boundaries.

### Implications

Our results identify that facial appearance is an important feature used in ascriptions of illegality, one that cannot be easily modified. This may be why illegalized immigrants enact alternative strategies for “legal passing” in daily life by carefully choosing modifiable cues: public apparel, behavior, speech^20^. The use of faces to ascribe illegality could play a role in why both illegalized immigrants and U.S. citizens who match the visual representation become targets of hate crimes and deportation procedures^89,90^.

Our results also speak to the role of language in perceptions of illegality. Research and immigrant activism on labeling effects suggest that “undocumented” is a strategically better term than “illegal” since it is less threatening^91,92^. In our results, “undocumented” was enough to visualize a racialized threat suggesting that both illegal/undocumented are too mired in criminalization to reclaim. Language activism may ultimately need to be (re)connected to broader struggles against racializing and criminalizing institutions^93^. For instance, using labels that productively redirect the origin of illegal status away from immigrants towards institutions that criminalize movement, such as *illegalized* immigrants^94^.

### Limitations

While we focused on representations of immigrant men from the perspective that they have been critical targets of the current deportation regime^66^, recent national conversations have centered around family and child separation at the border, highlighting how racist and gendered projects can shift. Broadening the demographic characteristics of the base face (e.g., women, children) could capture varied understandings of migrant illegality. A related limitation of this study is using the London Face Database to create the base face for studying U.S.-based migrant illegalization. Facial variation is distributed along geographic space^95^ and geography is one ingredient used for racialization^46,96^. Therefore, using faces more localized to the Americas in the base face could have captured more geographic variation in facial appearances within the representations. Including this regional face variation could influence the faces’ categorizations by better resembling the *presumed* appearances of categories associated with illegality (e.g., as a latinx or a Mexican person). Moreover, our race rating results relied on the average images, collecting race ratings of the individual images would provide better evidence if classification differences between documentation status representations are robust^60^.

Another potential limitation worth considering is whether representations of illegality may shift based on the racial composition of the face visualizers. For instance, racialized identity has been theorized to influence perceptions of immigrants^87^. Our MTurk sample was classified as majority white (52.7%), suggesting our pattern of results may reflect a white perceptual phenomenon. In line with this idea, an independent study using our materials with an all-white sample replicated the average citizen and immigrant face images found in our study^97^. However, against this idea, studies show that average analyses hide major perceptual disagreements about immigrants within racialized groups^98^. A separate study quantifying sources of heterogeneity in representations of illegalized immigrants^63^ found that variation was associated with visualizers’ ages and perceptions of local illegalized population sizes, rather than their own racialized identities. More studies with diverse populations are needed to carefully delineate the societal boundaries of the representational patterns we identified.

A last limitation involves the use of reverse correlation itself to study illegality. Despite attempts to use reverse correlation as simply another implicit measure that captures non-visual category associations^99^, its unique advantage for understanding discrimination lies in the ability to quantify *visual* associations. Returning to an earlier point, knowing someone associates illegality with Mexicans does not tell us which range of appearances and people they would visually classify as “illegal” (or even as Mexican) in social environments. Likewise, someone’s mental face representation does not tell us which categories they associate with illegality, the face could represent a wide range of nationalities or race categories. In this way, visual and non-visual methods are both necessary and complementary^31^. Reverse correlation images alone already contain valuable insights about discrimination that nuances information gained from non-visual methods. For instance, colorism and other featurisms stratify how people *within* a category experience discrimination^50^. Our results would suggest that (depending on the context) lighter-skinned people (Mexican or otherwise) may be less likely to be visually classified as “illegal” and experience resulting social consequences (even though lighter-skinned illegalized immigrants exist^31^). Unfortunately, efforts to understand these images often resort back to non-visual content analysis as we have done here (e.g., third-party raters evaluating the faces on various traits to more generally measure how the target category is evaluated). This practice transforms reverse correlation back into a method for assessing non-visual associations, which can be done much easier and more directly with other techniques like explicit self-report or shorter implicit association tasks^99^. More accessible and validated methods for analyzing meaningful features from *visual* data can help fully realize the advantages of reverse correlation.

## Conclusions

Given that movement is increasingly restricted in a globalized world, illegality continues to be forced upon migrating populations. This work reflects an initial examination into visual representations of illegalized immigrants. However, dimensions beyond visuality (e.g., linguistic, aural, geographic, material, cultural) must also be examined for understanding the continuing reification of illegality. Monitoring how these representations shift across time and geographies can provide a window into the way that imperial projects set the historical and ongoing conditions of possibility for specific conceptualizations of illegality to manifest or remain in the public mind^6,100^.

### Supplementary Information

Supplementary Information.

## Data Availability

All the data, analysis scripts, and preregistration are available on the Open Science Framework archive: https://osf.io/jzp3e/?view_only=68d3a6f348934be09a5412a1caab711d.
