# Supplementary material for: Immigration documentation statuses evoke racialized faceism in mental representations
Source: Sci Rep. 2024 May 9;14:10673. doi: 10.1038/s41598-024-61203-2 (PMC11082198; doi:10.1038/s41598-024-61203-2)
Supplement: Supplementary file 1 — Supplementary Information. [file 41598_2024_61203_MOESM1_ESM.pdf]

Immigration documentation statuses evoke racialized faceism in mental representations
Supplementary material

Table of contents:

- Appendix A. Differences from pre-registration
- Supplementary Figure S1. Comparisons between Princeton and Amazon Mechanical Turk (MTurk) samples' representations of undocumented immigrant
- Supplementary Figure S2. Average group-level CI trait ratings
- Supplementary Figure S3. Similarity Matrices.
- Supplementary Table S1. Participant demographics in Study 1, average face ratings
- Supplementary Table S2. Participant demographics in Study 1, individual face ratings
- Supplementary Table S3. Participant demographics in Study 2, similarity sorting task
- Supplementary Table S4. Correlations between similarity matrices.

Appendix

Appendix A. Differences from pre-registration

The overall project centered on a descriptive examination of representations, so the pre-registration was more general than what a confirmatory test would require. We list all deviations from the pre-registration in the followings:

- We stated that we would have the faces rated on how "foreign" they were perceived. After preregistering, we realized that this dimension could be unspecific in its referents (e.g., foreign from what vantage point and towards which direction?) and decided that "American" would be a more direct test of what we were interested in: inferences about their belongingness to the U.S. We did not collect ratings of "foreignness".
- We stated that we would collect 120 total participants in the image generating task (40 in each documentation status condition). Our final sample instead contained 50 participants in each condition, totaling up to 150 participants.
- We stated that we would exclude faces with an InfoVal of less than or equal to 1.56. An InfoVal is a numeric metric aimed at quantifying the informational value of a reverse correlation classification image (Brinkman et al., 2019). At the time of pre-registration, the InfoVal metric was yet to be published but reflected a novel and promising way to assess image reliability. However, while collecting data, we found that images that passed the 1.56 and more so the recommended 1.96 criterion were rare. We stopped data collection at 181 participants (N=59 citizen, N=70 documented, N=51 undocumented), and reassessed whether the exclusion criterion was too stringent. In order to ethically not discard a large amount of the data set that could be used, we decided to choose 50 faces from each condition from the data we had collected. These 50 faces would include those with the highest InfoVal scores, even if they were below criterion. So, the final sample included 150 total faces (50 from each condition), and through this procedure, 31 faces were excluded, those with the lowest InfoVal. Our difficulty obtaining high InfoVal-scoring faces reflect multiple possibilities. Maybe documentation statuses are not collectively solidified as visual stereotypes of gender categories are (gender was used to validate the InfoVal metric in Brinkman et. al. 2019). Another possibility is that our sample happened to collect unreliable data because of student characteristics or because our task was too difficult with 770 trials. In order to understand how our alternative exclusion procedure might have affected the resulting images, we created a classification image from only the faces that surpassed the InfoVal criterion in the undocumented immigrant condition (see image below). Both faces look nearly identical, suggesting that our average results in Study 1 would have been highly similar with the subset of only reliable images. This pattern suggests that the lower InfoVal-scoring images in our sample contribute little to the overall average. Lastly, this suggests that our individual classification face rating results in Study 1 are more conservative in that the results were obtained using individual face images that may have washed out any potential differences between conditions.

Final Sample
N = 50

High InfoVal Only
N = 25

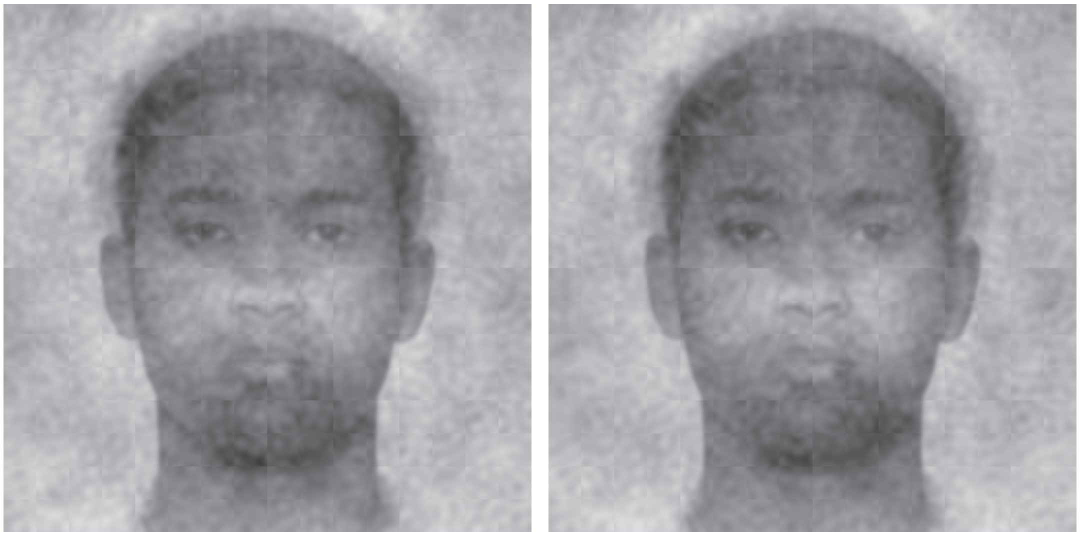

- We stated that the results of a power simulation with a hypothesized large effect size of *f*=.77 for the dangerous ratings that 20 participants would be enough for almost 100% power. Knowing that rating data from online samples is not always reliable, we stated that we would instead collect 40 participants per rating task in order to account for likely reliability exclusions. We achieved this sample size for the average classification image ratings which ended up exhibiting even larger effect sizes than .77. However, for the individual faces the sample sizes were often in the mid 30s, which is still above 20 participants, the minimum requirement estimated by our simulations. See figure below that tested how the number of participants affect power from the simulated model.

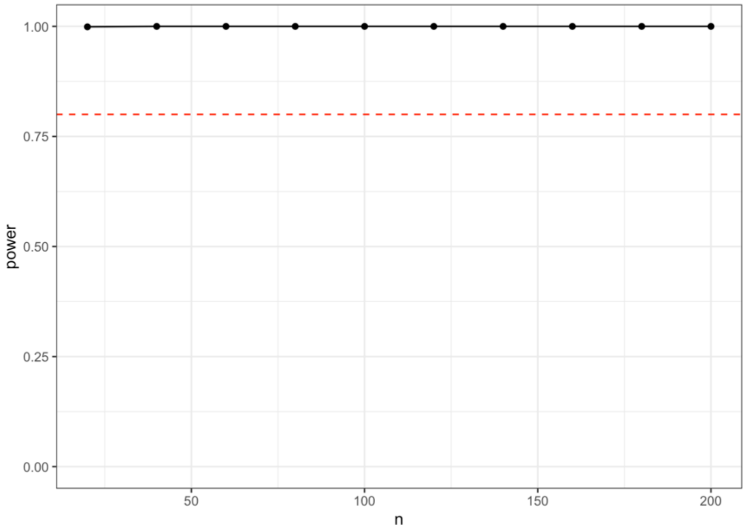

- We stated that we would collect 200 participants for the similarity sorting task, we instead collected 201 participants. The extra participant was a pilot participant to make sure the task worked. Since there were no issues, we decided to include them in the full sample.

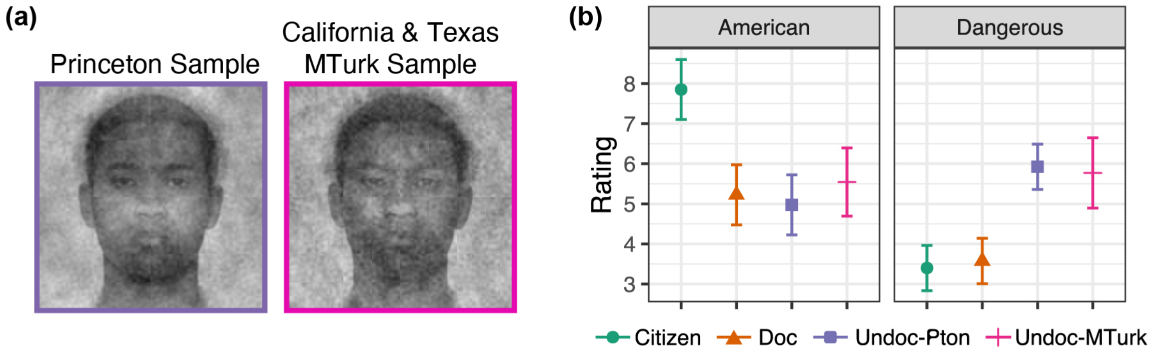

Supplementary Figure S1. Comparisons between Princeton and Amazon Mechanical Turk (MTurk) samples' representations of "undocumented immigrant". (a) The average classification image from a Princeton sample (N = 50) and an MTurk sample (N = 1002) using the same base face as Figure 1. The MTurk sample and data come from a separate paper (Martinez & Todorov, 2023). **(b)** Average American and dangerous ratings given to the average faces from this project (from Figure 2) and the average face from the MTurk sample (pink). Error bars represent 95% confidence intervals. The MTurk data were collected for a separate project and thus we only include measures from that project that overlap with this one (i.e., American and Dangerous ratings). The MTurk sample was located within California and Texas and thus represents representations from the U.S.-Mexico border, a geographically and socio-politically different area from the northeastern Princeton sample. Raters for the MTurk average face were also collected from MTurk (Dangerous N = 58, American N = 46). Participants completed 300 trials in the reverse correlation task with the same base face and stimulus pairs as this project. The MTurk average face was also ethnoracially classified (N = 87 MTurkers), however using a simple multiple forced choice measure rather than the likelihood measure from this study. The face was categorized as "black" by 82% of the sample followed by "Asian" by 9% of the sample.

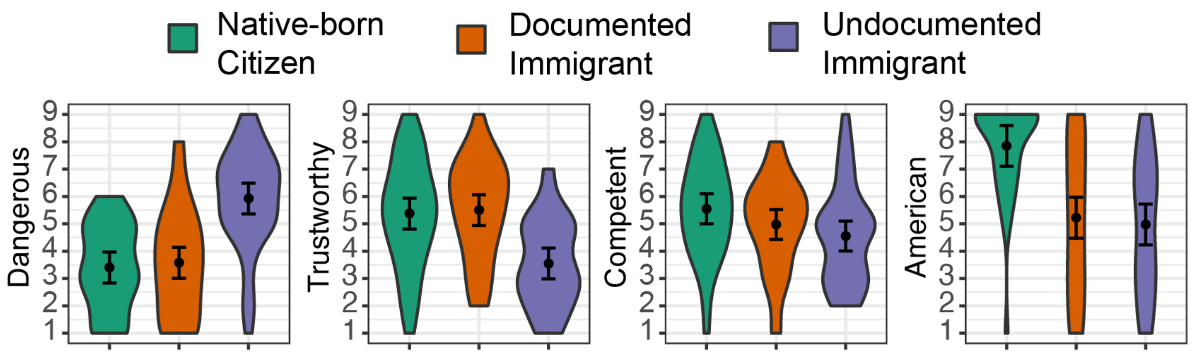

Supplementary Figure S2. Average group-level CI trait ratings. Ratings of average classification faces for native-born U.S. citizen (green), documented immigrant (orange), and undocumented immigrant conditions (purple). The violin shapes reflect the density of the rating data much like a sideways density plot. The error bars represent 95% confidence intervals.

Documentation status significantly predicted *American* (F(2,80)=29.6, p<.0001), *competent* (F(2,80)=4.1, p=.021), *dangerous* (F(2,80)=36.9, p<.0001), and *trustworthy* ratings (F(2,80)=25.1, p<.0001). The undocumented immigrant face was on average rated as less *American* (b=-2.88 CI[-3.88, -1.86], d=-1.22 CI[-1.61, -.84], t(80)= -6.94, p<.0001), *competent* (b=-1.0 CI[-1.86, -.13], d=-.58 CI[-.99, -.17], t(80)= -2.84, p=.017), *trustworthy* (b=-1.82 CI[-2.58, -1.07], d=-1.03 CI[-1.4, -.66], t(80)= -5.92, p<.0001), and more *dangerous* than the native born citizen face (b=2.52 CI[1.72, 3.33], d=1.42 CI[1.0, 1.83], t(80)= 7.68, p<.0001). The documented immigrant face was only significantly rated as less *American* than the native born citizen face (b=-2.65 CI[-3.64, -1.61], d=-1.12 CI[-1.5, -.74], t(80)= -6.34, p<.0001). The undocumented immigrant and documented immigrant faces were only significantly different in their perceived *dangerousness* (b=-2.35 CI[-3.15, -1.55], d=-1.32 CI[-1.73, -.92], t(80)= -7.16, p<.0001) and *trustworthiness* (b=1.95 CI[1.19, 2.70], d=1.10 CI[.73, 1.47], t(80)= 6.33, p<.0001). These results mirror the results from the ratings of the individual faces, but the effect sizes are magnified (i.e., inflated).

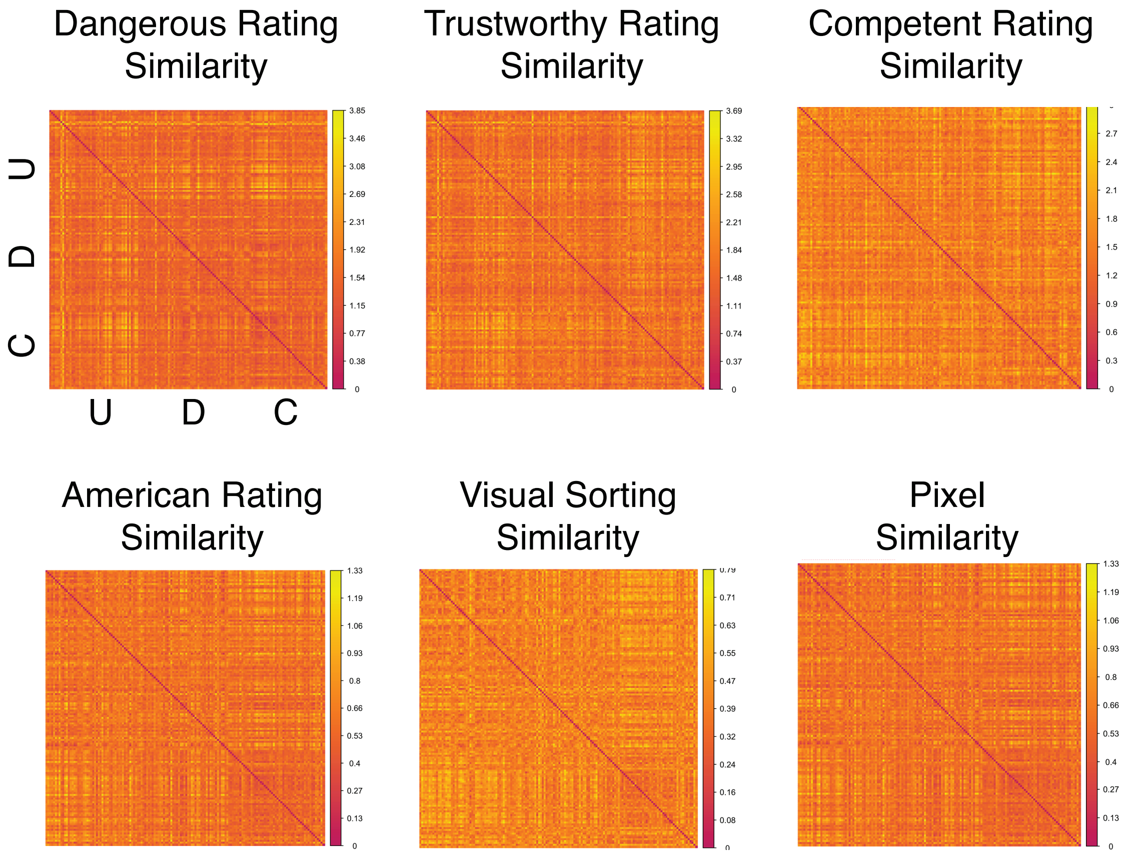

Supplementary Figure S3. Similarity matrices. Similarity matrices where the rows and columns represent all the individual classification images in top-down order from undocumented immigrant (U), documented immigrant (D), to citizen (C) as approximately depicted on the dangerous matrix. Darker red colors represent more similar faces, and lighter yellow colors are more dissimilar faces. Each matrix, except the pixel matrix, was created from separate samples who rated the same faces.

Supplementary Table S1. Participant demographics for Study 1, average face ratings

	American (N=40)	Competent (N=40)	Dangerous (N=40)	Race (N=40)	Trustworthy (N=40)
**Age**					
Mean (SD)	40.7 (13.2)	36.1 (10.6)	37.4 (12.1)	36.2 (9.62)	39.3 (11.6)
Median [Min, Max]	38.5 [24.0, 72.0]	33.0 [19.0, 68.0]	35.0 [22.0, 66.0]	35.0 [21.0, 64.0]	38.0 [18.0, 69.0]
**Gender**					
Female	19 (47.5%)	20 (50.0%)	17 (42.5%)	14 (35.0%)	16 (40.0%)
Male	21 (52.5%)	20 (50.0%)	23 (57.5%)	26 (65.0%)	24 (60.0%)
**Race**					
Asian/Pacific Islander	1 (2.5%)	1 (2.5%)	4 (10.0%)	3 (7.5%)	2 (5.0%)
Black or African	2 (5.0%)	3 (7.5%)	4 (10.0%)	2 (5.0%)	4 (10.0%)
Multiracial	4 (10.0%)	0 (0%)	2 (5.0%)	2 (5.0%)	3 (7.5%)
White or European	33 (82.5%)	33 (82.5%)	27 (67.5%)	31 (77.5%)	29 (72.5%)
Indigenous	0 (0%)	1 (2.5%)	0 (0%)	0 (0%)	0 (0%)
Latinx/o/a or Hispanic	0 (0%)	2 (5.0%)	3 (7.5%)	2 (5.0%)	2 (5.0%)
**Was born in U.S.**					
No	3 (7.5%)	2 (5.0%)	4 (10.0%)	3 (7.5%)	1 (2.5%)
Yes	37 (92.5%)	38 (95.0%)	36 (90.0%)	37 (92.5%)	39 (97.5%)
**Is U.S. citizen**					
No	1 (2.5%)	0 (0%)	0 (0%)	1 (2.5%)	1 (2.5%)
Yes	39 (97.5%)	40 (100%)	40 (100%)	39 (97.5%)	39 (97.5%)
**Live in U.S.**					
I am living in the U.S.	40 (100%)	40 (100%)	40 (100%)	39 (97.5%)	40 (100%)
I am living outside the US.	0 (0%)	0 (0%)	0 (0%)	1 (2.5%)	0 (0%)
**Immigrant Contact** [a]					
Mean (SD)	2.60 (1.46)	3.53 (2.00)	3.40 (2.01)	3.65 (1.90)	3.30 (1.86)
Median [Min, Max]	2.00 [1.00, 6.00]	3.00 [1.00, 7.00]	2.50 [1.00, 7.00]	3.00 [1.00, 7.00]	3.00 [1.00, 7.00]
**Undocumented Contact** [b]					
Mean (SD)	1.73 (1.06)	2.60 (1.63)	2.40 (1.91)	2.40 (1.53)	2.13 (1.30)
Median [Min, Max]	1.00 [1.00, 6.00]	2.00 [1.00, 7.00]	2.00 [1.00, 7.00]	2.00 [1.00, 7.00]	2.00 [1.00, 5.00]
**Social and Economic**					

	American (N=40)	Competen t (N=40)	Dangerous (N=40)	Race (N=40)	Trustwort hy (N=40)
**Conservatism Scale [c]**					
Mean (SD)	59.7 (18.4)	53.0 (20.0)	56.7 (19.5)	57.1 (13.6)	59.0 (17.8)
Median [Min, Max]	56.5 [24.1, 94.9]	53.6 [4.17, 89.7]	54.2 [19.2, 93.6]	54.8 [29.4, 83.3]	61.5 [11.4, 87.5]
**Social Dominance Orientation [d]**					
Mean (SD)	2.70 (1.53)	2.65 (1.64)	2.63 (1.72)	2.39 (1.31)	2.34 (1.28)
Median [Min, Max]	2.50 [1.00, 6.25]	2.31 [1.00, 7.00]	2.13 [1.00, 7.00]	2.13 [1.00, 6.88]	2.25 [1.00, 5.88]
Missing	0 (0%)	0 (0%)	1 (2.5%)	0 (0%)	3 (7.5%)

Note: Test-retest reliability was not calculated for the average face ratings as they were only rated once.

[a,b] Participants self-reported on a scale of 1 (never) to 7 (all the time) how often they are in contact with immigrants in general and with undocumented immigrants specifically on a daily basis.
[c] Participants responded whether they strongly oppose (1) or strongly favor (7) eight items related to preferences for group hierarchies (e.g., “Some groups of people are simply inferior to other groups.”) (Ho et al., 2015). Item scores are reverse-corrected and averaged to compute a single score where higher numbers reflect greater dominance orientation.
[d] The Social and Economic Conservatism Scale (SECS) was used to assess political orientation (Everett, 2013). The scale presents 12 political topics (e.g., military, abortion, gun ownership, patriotism). Participants respond on a temperature-like scale (0 (negative) to 100 (positive)) how they feel about each issue. Scores are reverse-corrected and averaged across topics to produce a final score where closer to 0 is more liberal and closer to 100 is more conservative.

Supplementary Table S2. Participant demographics in Study 1, individual face ratings

	Dangerous (N=37)	Competent (N=30)	Trustworthy (N=33)	American (N=35)
**Age**				
Mean (SD)	39.1 (11.4)	38.4 (11.2)	36.7 (13.1)	38.3 (14.0)
Median [Min, Max]	39.0 [3.00, 69.0]	36.0 [19.0, 63.0]	33.0 [22.0, 68.0]	32.0 [23.0, 71.0]
**Gender**				
Female	18 (48.6%)	14 (46.7%)	10 (30.3%)	20 (57.1%)
Male	19 (51.4%)	16 (53.3%)	22 (66.7%)	15 (42.9%)
Non-binary/Other	0 (0%)	0 (0%)	1 (3.0%)	0 (0%)
**Race**				
Asian/Pacific Islander	3 (8.1%)	0 (0%)	3 (9.1%)	3 (8.6%)
Black or African	3 (8.1%)	2 (6.7%)	3 (9.1%)	4 (11.4%)
White or European	31 (83.8%)	25 (83.3%)	26 (78.8%)	25 (71.4%)
Multiracial	0 (0%)	2 (6.7%)	1 (3.0%)	2 (5.7%)
Other	0 (0%)	1 (3.3%)	0 (0%)	0 (0%)
Latinx/o/a or Hispanic	0 (0%)	0 (0%)	0 (0%)	1 (2.9%)
**Was born in U.S.**				
No	1 (2.7%)	1 (3.3%)	1 (3.0%)	3 (8.6%)
Yes	36 (97.3%)	29 (96.7%)	32 (97.0%)	32 (91.4%)
**Is U.S. Citizen**				
Yes	37 (100%)	30 (100%)	33 (100%)	33 (94.3%)
No	0 (0%)	0 (0%)	0 (0%)	2 (5.7%)
**Lives in US**				
I am living in the U.S.	37 (100%)	30 (100%)	33 (100%)	35 (100%)
**Immigrant contact** [a]				
Mean (SD)	3.35 (1.96)	3.17 (1.76)	3.27 (1.59)	3.46 (2.01)
Median [Min, Max]	2.00 [1.00, 7.00]	3.00 [1.00, 7.00]	3.00 [1.00, 6.00]	3.00 [1.00, 7.00]
**Undocumented contact** [b]				
Mean (SD)	1.89 (1.02)	2.03 (1.52)	2.15 (1.37)	2.06 (1.21)
Median [Min, Max]	2.00 [1.00, 5.00]	2.00 [1.00, 7.00]	2.00 [1.00, 5.00]	2.00 [1.00, 6.00]
**Social and Economic Conservatism Scale** [c]				

	Dangerous (N=37)	Competent (N=30)	Trustworthy (N=33)	American (N=35)
Mean (SD)	57.6 (18.0)	59.0 (22.2)	55.0 (21.0)	58.9 (19.8)
Median [Min, Max]	55.5 [22.1, 90.1]	57.3 [12.5, 97.5]	50.3 [16.8, 92.3]	59.8 [18.1, 95.8]
**Social Dominance Orientation [d]**				
Mean (SD)	2.56 (1.58)	2.63 (1.71)	2.25 (1.43)	2.71 (1.52)
Median [Min, Max]	2.13 [1.00, 6.63]	2.25 [1.00, 6.25]	1.75 [1.00, 6.13]	2.50 [1.00, 6.13]
Missing	2 (5.4%)	1 (3.3%)	1 (3.0%)	1 (2.9%)
**Test-retest reliability**				
Mean (SD)	0.372 (0.191)	0.287 (0.171)	0.399 (0.245)	0.372 (0.217)
Median [Min, Max]	0.418 [~0, 0.724]	0.246 [0.0474, 0.658]	0.403 [0.00550, 0.797]	0.376 [0.0249, 0.803]

[a,b,c,d] See Supplementary Table S1 for more information on the tagged individual difference measures.

Supplementary Table S3. Participant demographics in Study 2, similarity sorting task

	Overall (N=201)
**Age**	
Mean (SD)	39.0 (11.4)
Median [Min, Max]	36.0 [20.0, 72.0]
**Gender**	
Female	103 (51.2%)
Male	96 (47.8%)
Other	2 (1.0%)
**Race**	
Asian/Pacific Islander	16 (8.0%)
Black or African	11 (5.5%)
Indigenous	1 (0.5%)
Latinx/o/a or Hispanic	4 (2.0%)
Multiracial	9 (4.5%)
Other	1 (0.5%)
White or European	159 (79.1%)
**Was born in U.S.**	
No	12 (6.0%)
Yes	189 (94.0%)
**Is U.S. citizen**	
No	3 (1.5%)
Yes	198 (98.5%)
**Lives in U.S.**	
I am living in the U.S.	199 (99.0%)
I am living outside the U.S.	2 (1.0%)
**Immigrant contact** [a]	
Mean (SD)	3.94 (1.93)
Median [Min, Max]	4.00 [1.00, 7.00]
**Undocumented contact** [b]	
Mean (SD)	2.35 (1.48)
Median [Min, Max]	2.00 [1.00, 7.00]
**Social and Economic Conservatism Scale** [c]	

	Overall (N=201)
Mean (SD)	57.6 (18.9)
Median [Min, Max]	56.8 [8.75, 100]
**Social Dominance Orientation [d]**	
Mean (SD)	2.27 (1.26)
Median [Min, Max]	1.88 [1.00, 6.63]

[a,b,c,d] See Supplementary Table S1 for more information on the tagged individual difference measures.

Supplementary Table S4. Correlations between similarity matrices.

Matrix pairs	Raw correlations			Partial Correlations		
	Lower CI	ρ	Upper CI	Lower CI	ρ	Upper CI
Visual-American	.48	.49	.51	.31	.33	.34
Visual-Dangerous	.25	.26	.28	.03	.05	.07
Visual-Trustworthy	.24	.26	.27	.01	.03	.05
Visual-Competent	.31	.33	.35	.07	.09	.11
Visual-Pixel	.36	.37	.39	.23	.25	.26
American-Dangerous	.32	.33	.35	.04	.06	.08
American-Trustworthy	.37	.38	.40	.15	.17	.18
American-Competent	.45	.46	.48	.25	.27	.29
American-Pixel	.31	.33	.34	.12	.14	.16
Dangerous-Trustworthy	.51	.52	.54	.41	.42	.44
Dangerous-Competent	.33	.35	.36	.10	.12	.13
Dangerous-Pixel	.19	.21	.23	.08	.10	.11
Trustworthy-Competent	.37	.39	.40	.16	.17	.19
Trustworthy-Pixel	.12	.14	.15	-.08	-.06	.-.04
Competent-Pixel	.21	.23	.24	.03	.05	.07

Note: bootstrapped confidence intervals for the direct correlations were computed using 5,000 bootstrap samples.
